# Hysterical Mania, Caused by Irritation of Spicula of Bone

**Published:** 1867-01

**Authors:** 


### SELECTED ARTICLES.

*Hyrsterical Mania, caused by Irritation of Spicula of Bone.*—At a recent meeting of the N. Y. Pathological Society, Dr. Sayre presented a portion of the calvarium and dura mater, removed by *post mortem* section from a lady twenty-five years of age, whom he had seen in consultation with Drs. Bloodgood and Brown-Séquard. He gave his testimony as follows: The patient, who was the mother of four children, had always enjoyed good health until her last sickness, and belonged to a healthy family. I first saw her on Sunday, June 3, she having on the Wednesday previous wakened out of a sound sleep with a most violent screaming delirium. Narcotics had been given her to make her tranquil, but nothing but choroform had been of any avail. If the administration of the anæsthetic was not kept up she would arouse herself, and shriek to the top of her voice, seemingly furious over a pain in the top of her head and left temple. There had been no excitement of the pulse during all this time, and her skin seemed to be natural; there was no coating on her tongue. Her bowels were regular, and her kidneys had acted well until that day (June 3), when a catheter was called into requsition. This instrument had afterwards to be constantly used until her death. A careful examination of the urine disclosed nothing abnormal. Finding no cause for the difficulty, I pronounced the case one of hysteria. An injection of ten drops of Magendie's solution was given, and the result was, that in five or ten minutes she was asleep. Being out of town at the time, the patient felt so much better, that the following morning she came to the city. In a few hours after her arrival she had another attack, and I was immediately sent for. I found her in the most violent contortinos, seemingly wild with the pain in her head, and shrieking so as to alarm the neighborhood. When you spoke sharply to her you could control her to some extent for a time, but any moment she would "go off" into the most violent contortions of her body, and scream as before. Dr. Brown-Séquard then saw the case with me, and pronounced it one of hysterical mania. The frequency and severity of the spasms seemed to increase upon her for two or three days, when I observed that she was reckless in regard to her striking out; and finding that she had bruised herself seriously in consequence, I began to suspect that there was not so much of an hysterical element in the disease as had at first been supposed. We found that nothing afforded her even temporary relief, except chloroform by inhalation. She inhaled eight pounds of the anæsthetic from the 3d to the 16th of June, during most of which time it had been incessantly administered to her. Dr. Bulkley saw her in consultation, and he continued to visit her twice a day until she died. Her stomach could not retain anything fluid, or solid, and after a while she persisted in refraining from all nourishment. She had a tendency to lie upon her face, with her head buried in the pillow, her legs being kept in a perpetual and violent motion. Notwithstanding this severe tax upon her vital powers, she did not seem to waste away any, and her muscles were possessed of their usual hardness, and her body of its customary plumpness. After a time the urine, which was by the way copious enough, began to give forth a most sickening odor, and finally became so intolerable that at the time it was drawn off the other occupants of the room would actually be driven out. This strange and disagreeable odor we were disposed to refer to the large quantity of chloroform inhaled. Her menstruation came on about the 9th instant, and continued to be normal until the 14th, when it ceased. On the latter day there was noticed a marked weakness in one of the lower extremities, and on the day following this amounted to complete paralysis. The succeeding day the other leg became similarly affected, and that evening she died.

*Autopsy.*—The genital organs were perfectly normal. The abdomen was perfectly healthy, although the cavity of the peritoneum was entirely dry. The intestines were healthy, and the kidneys normal; but the liver was considerably enlarged, was of an ochrey color, and so friable that it fell to pieces in the attempt to remove it. The heart and lungs were healthy. On opening the brain there was a good deal of congestion; there was, however, no apoplectic effusion, and the brain itself was very much more than ordinarily firm. On the left side of the dura mater there was quite a sharpened projection of bone, about a quarter of an inch in length, and also a similar piece in the situation of the falx cerebri, as well as smaller and more rounded pieces from other portions of the membrane. The first spiculum discovered was, on the point projecting against the brain, as sharp as a needle. This was all that could be discovered about her. I am disposed to think that the irritation caused by these spicula gave rise to the patient's disease. I am as confident as I can be that she suffered from great pain in these situations, as she would constantly point with her fingers to these two spots, on the top and side of her head, and entreat us in the wildest sort of desperation to "bore a hole" there, and relieve her.

Dr. Rogers stated that it was not uncommon to find acute softening of the liver in cases that proved rapidly fatal. He suggested that the chloroform might have had something to do in bringing about this result.

Dr. Hamilton thought that there might be a possibility that the true cause of the trouble was not found after all, as it was very natural at first sight to refer such symptoms to a piece of projecting bone, when in reality the deposit might have had little or nothing to do with it.

Dr. Sayre remarked that the patient, for some months previous to death, had been subject to melancholia; had anticipated death with such certainty that she could not be persuaded out of it, and had gone to the trouble of having a number of photographs taken for such of her friends as she wished to be remembered by. He was of the opinion that the portions of bone which projected into the cavity of the cranium, were at the time not large enough to produce any other brain symptoms, but that afterwards growing more rapidly, violent convulsive actions resulted.

Dr. Post did not think that the projections were large enough to injure the brain by pressure, but believed that the friction of the organ against the sharp points gave rise to the irritation.

